# The use of ethanol as contrast enhancer in synchrotron X-ray phase-contrast imaging leads to heterogeneous myocardial tissue shrinkage: a case report

**DOI:** 10.1107/S1600577524010221

**Published:** 2025-01-01

**Authors:** Gabriel Bernardino, Àngels Calvet-Mirabent, Hector Dejea, Eduard Guasch, Anne Bonnin, Patricia Garcia-Canadilla

**Affiliations:** a BCN MedTech Universitat Pompeu Fabra Barcelona Spain; b Institute of neurosciences, Department of Medicine, School of Medicine and Health Sciences University of Barcelona Barcelona Spain; c European Synchrotron Radiation Facility Grenoble France; d Institute of Cardiovascular Science University College London London United Kingdom; e Institut d’Investigacions Biomèdiques August Pi i Sunyer (IDIBAPS), Barcelona, Spain; f Arrhythmia Unit, Department of Cardiology, Hospital Clínic de Barcelona, Barcelona, Spain; g Centre for Biomedical Research on Rare Diseases (CIBER-ER), Hospital Clínic de Barcelona, Barcelona, Spain; h Paul Scherrer Institut Villigen PSI Switzerland; i Cardiovascular Resarch Group iCare4Kids, Institut de Recerca Sant Joan de Déu, Esplugues de Llobregat, Spain; Paul Scherrer Institute, Switzerland

**Keywords:** synchrotron imaging, micro-CT, heart, ethanol, myocardial architecture, cardiomyocyte, image contrast, synchrotron phase-contrast imaging

## Abstract

One of the main limitations of conventional absorption-based X-ray micro-computed tomography imaging of biological samples is the low inherent X-ray contrast of soft tissue. To overcome this limitation, the use of ethanol as contrast agent has been proposed to enhance image contrast of soft tissues through dehydration. Some authors have shown that ethanol shrinks and hardens the tissue too much, also causing small tissue ruptures due to fast dehydration. However, the local tissue deformation occurring as a consequence of tissue dehydration and whether tissue shrinkage can modify myocardial architecture has not been quantified yet. The aim of this paper is to quantify the local myocardial tissue deformation due to ethanol dehydration based on 3D non-rigid registration and perform a detailed characterization of its myocardial tissue organization, before and after ethanol dehydration. A rat adult heart was imaged with synchrotron-radiation-based X-ray phase contrast imaging (X-PCI) three times: before, 9 h after and 342 h after ethanol immersion. The total volume shrinkage as well as changes in the left ventricular myocardial thickness were computed. Then, to determine local deformation of the heart caused by ethanol dehydration, the related 3D tomographic datasets were registered by means of a non-rigid registration algorithm. Finally, changes on the orientation and organization of myocytes were assessed. Our results show that the use of ethanol in synchrotron X-PCI can improve image contrast, but the tissue shrinkage is not homogeneous thus changing the local myocardial organization.

## Abstract 2

In this work, we show that the use of ethanol to increase image contrast when imaging cardiac tissue with synchrotron X-ray phase-contrast imaging leads to heterogeneous tissue shrinkage, which has an impact on the 3D organization of the myocardium.

## 1. Introduction

The study of the macro- and micro-structure of cardiac tissue is essential for a better understanding of the cardiac structure and function relationship, both in healthy and under pathological conditions. To date, conventional histology remains the gold standard for the analysis of cardiac tissue microstructure. Although being very powerful in terms of contrast and resolution, it involves several processes such as tissue dehydration, slicing and staining thus altering tissue native structure. In conventional histology, tissue sections are individually visualized under a microscope resulting in two-dimensional (2D) microscopic images. The possibility of reconstructing an entire volume from serial 2D slices has been extensively investigated, but still remains very challenging (Pichat *et al.*, 2018 ▸).

Three-dimensional (3D) high-resolution techniques such as X-ray microcomputed tomography (micro-CT) have emerged as a powerful tool for 3D imaging of biological samples with high-resolution (<1 µm voxel size) and allowing the production of virtual tissue slices without destroying the sample, and is therefore sometimes referred to as virtual histology (Bournonville *et al.*, 2019 ▸; Busse *et al.*, 2018 ▸; Albers *et al.*, 2018 ▸). Moreover, with the development of synchrotron facilities, more brilliant X-ray sources are available, thus improving image quality and resolution and significantly reducing the scanning time. However, one of the main limitations of absorption-based X-ray micro-CT imaging of biological samples is the low absorption of soft tissue. To overcome this limitation several different approaches have been proposed, from the use of different contrast agents such as iodine or phospho­tungstic acid (PTA) that bind to the tissue of interest, hence increasing its X-ray attenuation coefficient (Bournonville *et al.*, 2019 ▸; Silva *et al.*, 2015 ▸; Koç *et al.*, 2019 ▸; Metscher, 2009 ▸; O *et al.*, 2021 ▸), to the use of other imaging modalities such as X-ray phase-contrast imaging (X-PCI) that uses the refractive properties of brilliant X-rays to enhance soft tissue image contrast (Bravin *et al.*, 2013 ▸). A variety of X-PCI methods including grating or crystal interferometry, analyzers or propagation-based (PB) are currently available (Bravin *et al.*, 2013 ▸). Among these methods, PB X-PCI is the simplest solution in terms of experimental setup and has proven to be very powerful for the structural examination of cardiac tissue (Reichardt *et al.*, 2020 ▸; Reichardt *et al.*, 2020 ▸; Varray *et al.*, 2017 ▸; Mirea *et al.*, 2015 ▸; Wang *et al.*, 2019 ▸; Gonzalez-Tendero *et al.*, 2017 ▸; Dejea *et al.*, 2019 ▸; Soveral *et al.*, 2020 ▸; Loncaric *et al.*, 2021 ▸; Garcia-Canadilla *et al.*, 2018 ▸; Planinc *et al.*, 2021 ▸).

However, although soft tissue contrast is highly increased in X-PCI, several authors have proposed the use of ethanol to increase image contrast even further (Reichardt *et al.*, 2020*a* ▸; Reichardt *et al.*, 2020*b* ▸; Varray *et al.*, 2017 ▸; Mirea *et al.*, 2015 ▸; Patzelt *et al.*, 2019 ▸; Mrzílková *et al.*, 2019 ▸; Dudak *et al.*, 2016 ▸; Shirai *et al.*, 2014 ▸; Takeda *et al.*, 2014 ▸; Takeda *et al.*, 2013*a* ▸; Lwin *et al.*, 2022 ▸; Kunii *et al.*, 2013 ▸; Takeda *et al.*, 2013*b* ▸). Ethanol is commonly used for tissue fixation (Patzelt *et al.*, 2019 ▸; Haque *et al.*, 2020 ▸; Essen *et al.*, 2010 ▸) as well as a contrast agent both in conventional (tube-based) and synchrotron-based micro-CT imaging (Reichardt *et al.*, 2020*a* ▸; Reichardt *et al.*, 2020*b* ▸; Varray *et al.*, 2017 ▸; Mirea *et al.*, 2015 ▸; Shirai *et al.*, 2014 ▸; Kunii *et al.*, 2013 ▸; Takeda *et al.*, 2013*b* ▸). However, despite the increase in image contrast, several studies have reported significant tissue shrinkage due to ethanol dehydration as well as small tissue ruptures due to fast dehydration (Patzelt *et al.*, 2019 ▸; Dudak *et al.*, 2016 ▸; Hołda *et al.*, 2016 ▸; Hoshino *et al.*, 2014 ▸; Buytaert *et al.*, 2014 ▸; Vickerton *et al.*, 2013 ▸). Most of these studies have only quantified tissue shrinkage globally, without evaluating whether the tissue shrinks homogeneously or not, and how this affects the myocardial organization.

Cardiovascular disease is the leading cause of death in Europe, with over 3.9 million deaths (ESC, 2019 ▸). It involves cardiac remodelling at the cellular, tissue and organ level. Cardiomyocytes, which are the primary functional muscle cells in the myocardium, are aggregated and aligned in a pre-dominant direction within the myocardium, forming a complex 3D mesh. This complex organization of cardiomyocytes within the myocardium determines the propagation of electrical waves and force development within cardiac tissue. Therefore, a comprehensive structural assessment of cardiac remodelling at different length scales is needed in order to better understand how changes in cardiac structure affect cardiac function in cardiovascular disease. Synchrotron-based X-PCI has proved to be a powerful tool for the study of structural changes of hearts from rabbit fetuses (Gonzalez-Tendero *et al.*, 2017 ▸; Garcia-Canadilla *et al.*, 2019 ▸), young rats (Planinc *et al.*, 2021 ▸; Dejea *et al.*, 2019 ▸), human fetuses (Garcia-Canadilla *et al.*, 2018 ▸) as well as human endomyocardial biopsies (Planinc *et al.*, 2023 ▸; Loncaric *et al.*, 2021 ▸) at micrometre resolution with sufficient contrast to distinguish myocytes at the cellular level and without the need for destructive sectioning or tissue processing. However, in order to properly quantify myocardial microstructure, especially cardiomyocytes aggregates (often referred to as myofibers and sheets/sheetlets), it is crucial to minimize structural changes in cardiac tissue as a consequence of the imaging process.

The aim of this study is to evaluate the local myocardial tissue deformation due to ethanol dehydration based on 3D non-rigid registration of synchrotron-based X-PCI images of a rat heart and detailed characterization of its myocardial tissue organization, before and after ethanol dehydration.

## 2. Materials and methods

### 2.1. Sample preparation

One 1.5-month-old male Wistar rat was obtained from Charles River Laboratories. The rat was housed individually and maintained at 21°C with a 12 h day/night cycle. Food and water were administrated *ad libitum*. The rat was sacrificed after two weeks, the heart was rapidly excised, rinsed in PBS + 2% heparin solution and immersed in 4% paraformaldehyde. Animal care and experimentation conformed to the European Union (Directive 2010/63/UE) and Spanish guidelines (RD 53/2013) for the use of experimental animals. Approval was obtained from the local animal research ethics committee ‘Comité de Ética de Experimentación Animal (CEEA)’ (CEEA 68/5435).

For the image acquisition, the heart was placed in a cylindrical plastic holder filled with degassed deionized water to hold the heart in place without compression and avoid motion artefacts during tomography acquisition. The heart was first scanned with synchrotron PB X-PCI without further sample preparation. Then, the heart was immersed in successive baths (lasting 30 min) increasing the ethanol concentration from 10% to 70% in 10% steps as described by Varray *et al.* (2017 ▸, 2013 ▸). After that, the heart was scanned again. Then the heart was kept in 70% ethanol for 12 days and subsequently the third and last scan was performed. Therefore, the sample was scanned three times: before, 9 h after and 342 h after ethanol immersion. All three scans were performed with the same PB X-PCI setup and acquisition parameters.

### 2.2. Image acquisition

The heart was scanned with synchrotron PB X-PCI at the TOMCAT beamline (X02DA) of the Swiss Light Source (Paul Scherrer Institute, Switzerland). The plastic tube containing the heart was placed on the rotation stage located 333 cm from the detector. A 20 keV parallel synchrotron X-ray beam (monochromaticity bandwidth of 2%) was used to image the sample with 5.8 µm effective pixel size and field of view (FoV) of 11.83 mm × 3.29 mm. Acquisition was performed by rotating the sample through 360° acquiring 2501 projections (exposure time: 20 ms). X-rays were converted to visible light through a LuAG:Ce 300 µm scintillator and detected by an sCMOS camera (PCO.Edge 4.2). Since the heart was larger than the FoV, several overlapping scans from base to apex were performed to fully cover the full height. Additionally, two series of 50 flat and 20 dark images were acquired for flat-field and dark-field corrections of each acquisition. The acquired projections were reconstructed using the Gridrec algorithm (Marone & Stampanoni, 2012 ▸). All datasets were reconstructed with the same contrast limits. Finally, the reconstructed datasets were stitched in order to obtain a single full heart dataset.

### 2.3. Image analysis

#### 2.3.1. Image alignment

A multi-resolution rigid registration between all image datasets was performed in order to correct for differences in sample position during scanning. Briefly, the rigid transformation that minimized the Matte’s mutual information (MMI) (Mattes *et al.*, 2001 ▸) between moving (9 h and 342 h after ethanol immersion) and reference (before ethanol) image datasets was found. To do that, a multi-resolution framework consisting of three stages was used, in which the resolution of the original image was successively reduced with respective subsampling factors of 4, 8 and 16. The minimization problem was solved via the limited Broyden–Fletcher–Goldfarb–Shanno algorithm (L-BFGS) optimization method (Mattes *et al.*, 2001 ▸). Therefore, both after ethanol immersion datasets were transformed to be aligned to the reference dataset.

#### 2.3.2. Cardiac tissue segmentation

Cardiac tissue was semi-automatically segmented using the two-stage pixel classification module from the open-source software *Ilastik* (Berg *et al.*, 2019 ▸; Sommer *et al.*, 2011 ▸). Then, different morphological operations were applied to smooth the segmentation using an in-house algorithm implemented in MATLAB. Finally, blood clots inside the ventricular cavities were manually removed using *Seg3D* software (Cibc, 2013 ▸).

#### 2.3.3. Quantification of image contrast and quality

**Figure 1:**
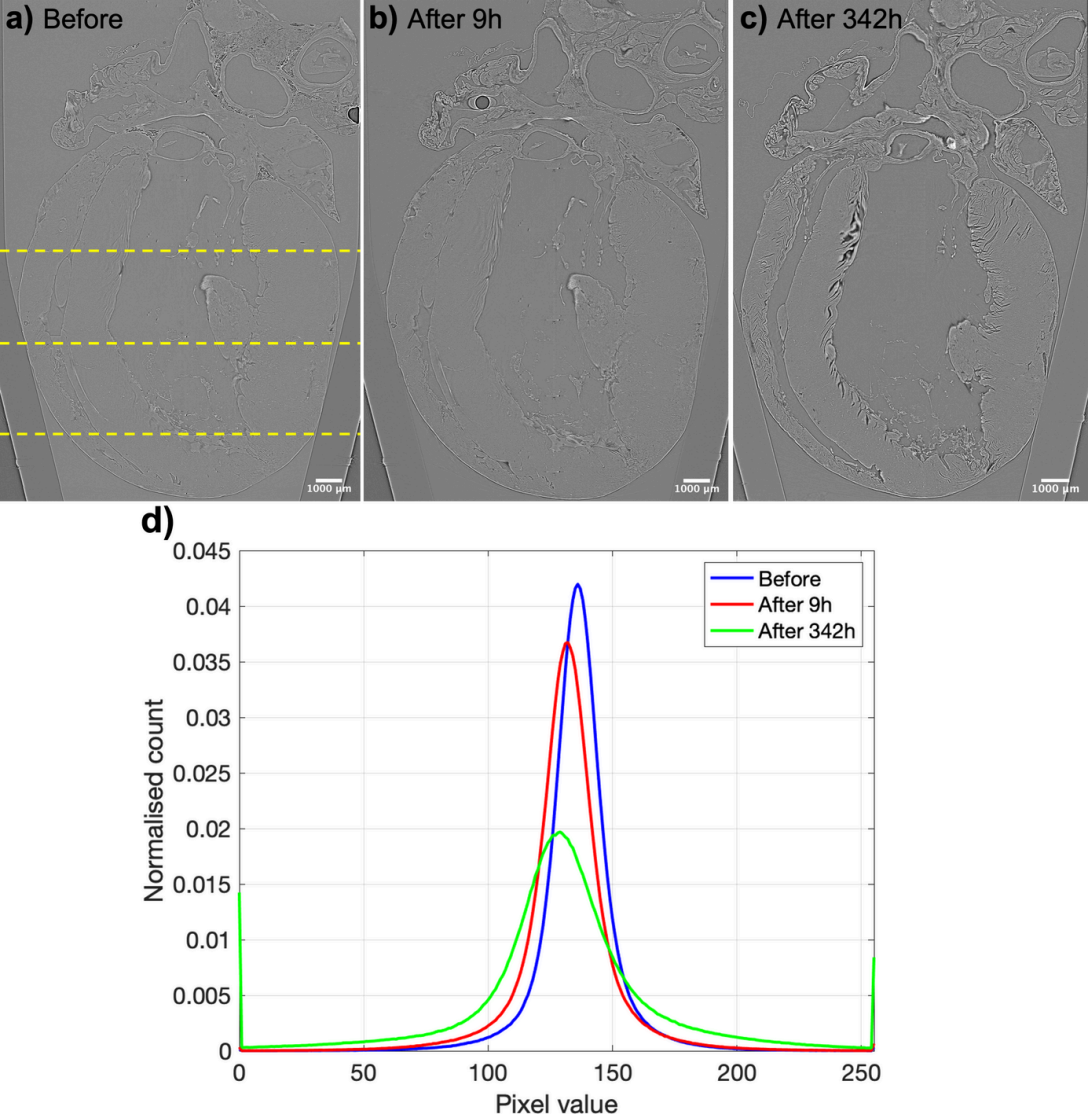
Demonstration of the contrast improvement in synchrotron X-PCI and sample shrinkage due to ethanol dehydration between (*a*) native heart kept in formalin, (*b*) 9 h after and (*b*) 342 h after immersion in 70% ethanol solution. (*d*) Distribution of pixel intensities in the three datasets. All the datasets were reconstructed using the same contrast limits. Dashed lines in panel (*a*) indicate the three apico-basal slices selected for quantification.

The parameters used to evaluate the differences in image contrast and quality due to ethanol dehydration were the tissue contrast (*C*), the contrast-to-noise ratio (CNR) (Brombal *et al.*, 2019 ▸, 2018 ▸) and the signal-to-noise ratio (SNR) as follows,
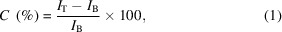

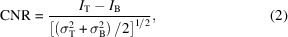

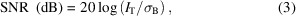
where subscripts T and B denote the cardiac tissue and background, respectively, and *I*_*i*_ and σ_*i*_ are the mean and standard deviation voxel intensity values, respectively. These parameters were computed in three different image slices from apex to base (see Fig. 1 ▸) for each dataset. Finally, distributions of the voxel intensity values within the cardiac tissue were also computed.

#### 2.3.4. Image non-rigid registration

To evaluate and quantify the tissue shrinkage produced by ethanol dehydration and study whether the deformation was uniform, a 3D non-rigid registration between the before (fixed) and 342 h after ethanol (moving) datasets was performed to obtain the local deformation field that warped the moving towards the fixed dataset. The moving dataset was previously aligned to the fixed images using the rigid registration described previously.

The symmetric force additive demons algorithm, which is a variation of the classical demons algorithm in which the forces are symmetric (Wang *et al.*, 2005 ▸; Rogelj & Kovačič, 2006 ▸), was used for computing the transformation that wraps the moving to fixed image. The demons algorithm assumes that the intensity of homologous points of moving and fixed images are equal. However, this assumption is not valid since the image intensity changes among the different datasets due to the use of ethanol. Therefore, we used histogram equalization prior to the non-rigid registration in order to create a more exact match between the intensities of the two datasets. Due to the large size of the original datasets (4080 × 4080 × 3747) we used a multi-resolution pyramids approach, in which the resolution of the original image was successively reduced, consisting of three stages with respective down-sampling factors of 4, 8 and 16. The implementation of the symmetric demons algorithm available in *Simple ITK* was used (Lowekamp *et al.*, 2013 ▸; Yaniv *et al.*, 2018 ▸; Beare *et al.*, 2018 ▸). A more detailed explanation of the non-rigid registration method can be found in Section S1 of the supporting information.

*Full resolution non-rigid registration in a region of interest.* In order to study the local tissue shrinkage produced by ethanol dehydration in more detail, a region of interest (ROI) of 150 × 150 × 150 pixels within the left ventricle (LV) was manually selected, in which the non-rigid registration was refined. To do so, a second non-rigid registration between the fixed (before ethanol) and moving (342 h after ethanol non-rigid registered) ROIs at full resolution was carried out using the same symmetric demons algorithm. The size of the ROI was chosen in order to be able to quantify changes in cardiac tissue at full resolution without prohibitive computational cost.

*Evaluation of registration performance.* The Dice similarity coefficient between the reference and registered datasets was computed to quantitatively evaluate the quality of the registrations. The Dice coefficient was calculated as
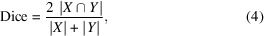
where |*X* ∩ *Y*| represents the common voxels between both datasets, and |*X*| and |*Y*| represent the number of voxels in *X* and *Y*, respectively.

#### 2.3.5. Quantification of global and local tissue deformation

To quantify global tissue shrinkage due to ethanol dehydration, the myocardial volume of all three aligned datasets was computed. Then, the LV wall thickness was automatically measured in the anterior, posterior septal and lateral walls in three apico-basal image slices (see Fig. 1 ▸).

Finally, the non-rigid registration was then used to recover the deformation fields and quantify the local tissue shrinkage produced by ethanol dehydration. To do that, the Jacobian determinant, *J*_D_, of the deformation field was calculated. In particular, *J*_D_ measures how the voxel volume changes after non-rigid registration, where *J*_D_ > 1.0 denotes volume dilation while *J*_D_ < 1.0 means volume shrinkage.

#### 2.3.6. Analysis of myocardial organization

Myocardial organization was assessed by means of a structure tensor (ST) based method implemented in MATLAB as described in the literature (Gonzalez-Tendero *et al.*, 2017 ▸; Garcia-Canadilla *et al.*, 2018 ▸; Baličević *et al.*, 2015 ▸), in all three datasets: before, 9 h after and 342 h after ethanol immersion. Briefly, the image intensity gradient in the *x*, *y* and *z* directions was obtained for each voxel using a central difference algorithm within the voxel’s cubical neighbourhood. The ST was then calculated as the cross-product of gradient vectors. Then, eigen-decomposition of the ST was performed thus obtaining the three eigenvalues (λ_*i*_) and eigenvectors (**v**_*i*_).

Different parameters based on the relative magnitude of the eigenvalues were calculated to quantify the anisotropy of myocardial tissue. In a manner analogous to diffusion tensor imaging (DTI), fractional anisotropy of the structure tensor, FA_ST_, is defined as
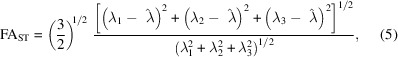
where 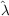 = 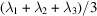 is the average of the tensor’s eigenvalues. In the same manner, the spherical (*C*_s_), linear (*C*_l_) and planar anisotropy (*C*_p_) are defined (Nelson *et al.*, 2018 ▸),
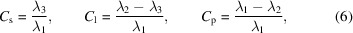
with *C*_s_ + *C*_l_ + *C*_p_ = 1, and 0 ≤ *C*_s_, *C*_l_, *C*_p_ ≤ 1.

Then, the eigenvector with the smallest eigenvalue (λ_3_), known as the tertiary eigenvector (**v**_3_), was selected as the vector pointing in the main direction of the myocyte aggregates. In order to assess the orientation of the myocyte aggregates, its helical angle (HA), defined as the angle between the tertiary eigenvector **v**_3_ and the transverse plane defined by the local circumferential and radial directions of the cylindrical coordinate system of the heart, was calculated. All the quantitative parameters (FA_ST_, *C*_s_, *C*_l_, *C*_p_ and HA) were calculated in the rigidly aligned datasets.

*Quantification of myocyte aggregates orientation.* The myocyte aggregates orientation was quantified in the same three apico-basal image slices selected to quantify image contrast and quality (see Fig. 1 ▸). In order to quantify the changes in myocardial organization induced by ethanol dehydration, the calculated deformation fields of the non-rigid registrations between the after and before ethanol immersion datasets obtained in Section 2.3.4 were applied to all parameters calculated (FA_ST_, *C*_s_, *C*_l_, *C*_p_ and HA) of the moving datasets. Distributions of FA_ST_, *C*_s_, *C*_l_, *C*_p_ and HA within the cardiac tissue at each apico-basal image slice were computed.

Finally, transmural profiles of HA from endo- to epicardium were obtained for four different ROIs within the LV: anterior, lateral, posterior and septal walls, and for each of the three apico-basal image slices. Next, a linear regression fitting,*y* = β_1_*x* + β_0_, was applied to all transmural HA profiles. The *R*^2^ coefficient, of the linear fitting, which characterizes the linearity of the profiles, was also computed.

### 2.4. Statistical analysis

Normality of data was tested with a Shapiro–Wilks test, adequate for a small number of samples. Normally distributed variables were expressed as mean ± standard deviation while non-normally distributed continuous variables were expressed as medians with interquartile ranges.

## 3. Results

### 3.1. Quantification of image contrast and quality

After 9 h of ethanol treatment, the tissue contrast (*C*) was slightly improved, from 1.08% to 2.80%, also demonstrated by the increase in CNR (see Table 1 ▸) without significant changes in image quality. After 342 h of ethanol immersion, the tissue contrast was further improved up to 3.88%, as clearly noticed also in the raw X-PCI images [Figs. 1 ▸(*a*)–1(*c*)], which is also demonstrated by the wider distribution of the voxel intensities [see Fig. 1 ▸(*d*)]. However, the image quality was slightly reduced by 3 dB, explained by the higher amount of image artefacts resulting in an increase of the background’s standard deviation (σ_B_).

### 3.2. Quantification of global and local tissue shrinkage

**Figure 2:**
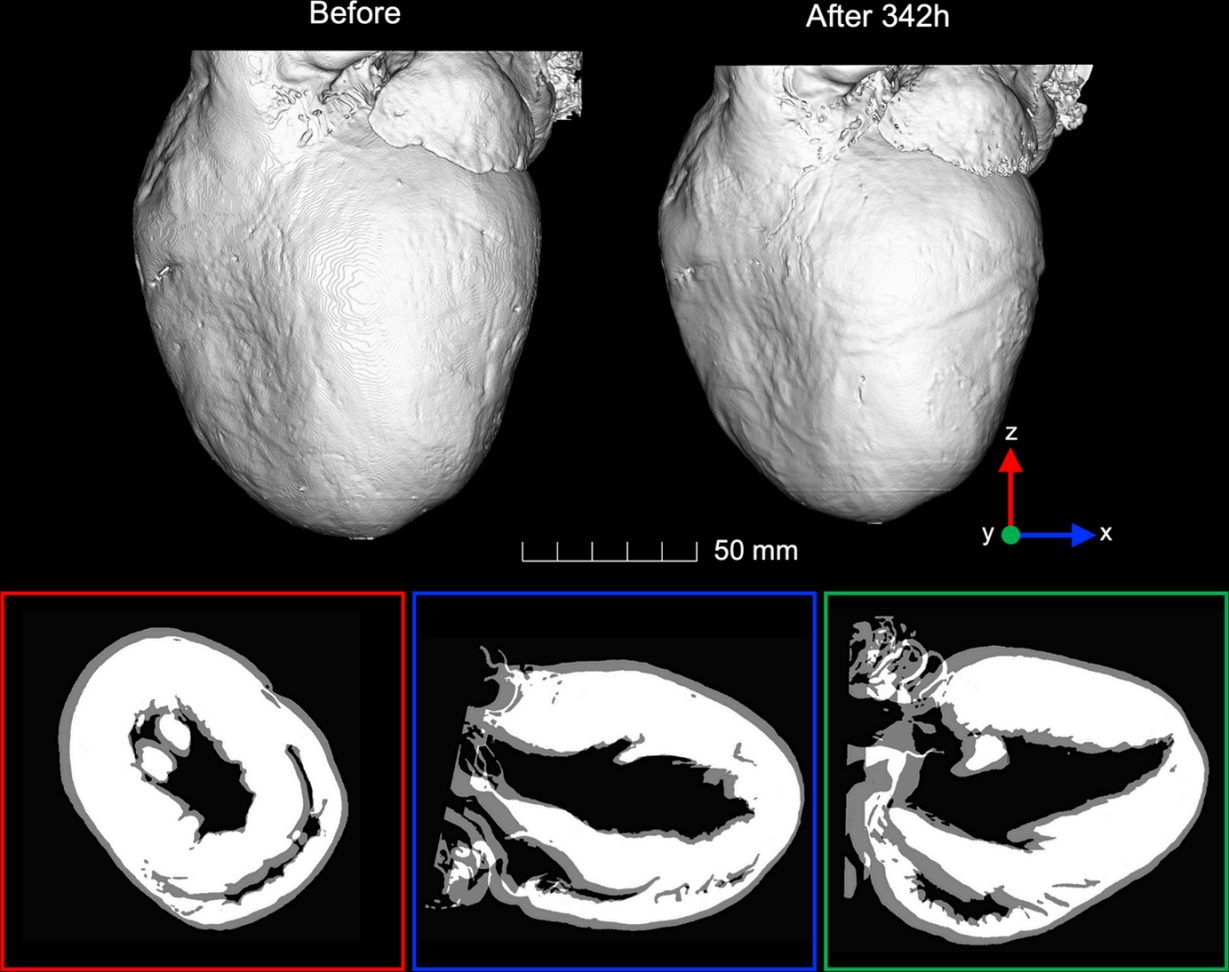
Top: three-dimensional reconstruction of the rat heart before and 342 h after 70% ethanol immersion rigidly aligned. Bottom: 2D resliced images along the three orthogonal planes *x* (blue), *y* (green) and *z* (red), clearly showing the good alignment between both datasets and the volume shrinkage of the heart after long-term immersion in ethanol.

We found that long-term immersion (342 h) of the heart in ethanol led to a significant reduction of myocardial volume of −27.7% (from 0.75 cm^3^ to 0.54 cm^3^) due to dehydration (Table 2 ▸, Fig. 2 ▸). Moreover, long-term immersion in ethanol also resulted in a heterogeneous reduction of LV myocardial thickness of about −18.33% on average, being more pronounced in the basal and mid-anterior and lateral walls (Table 2 ▸).

Regarding the local deformation, results of the non-rigid registration between the before and 342 h after ethanol immersion datasets can be found in Figs. S1 and S2. A Dice similarity coefficient of 0.97 demonstrates the good matching between reference and registered datasets, which is also illustrated in Fig. S1. When performing a finer registration in a ROI (Fig. S2), the Dice coefficient was 0.99, thus demonstrating an improvement of the previous coarser non-rigid registration.

**Figure 3:**
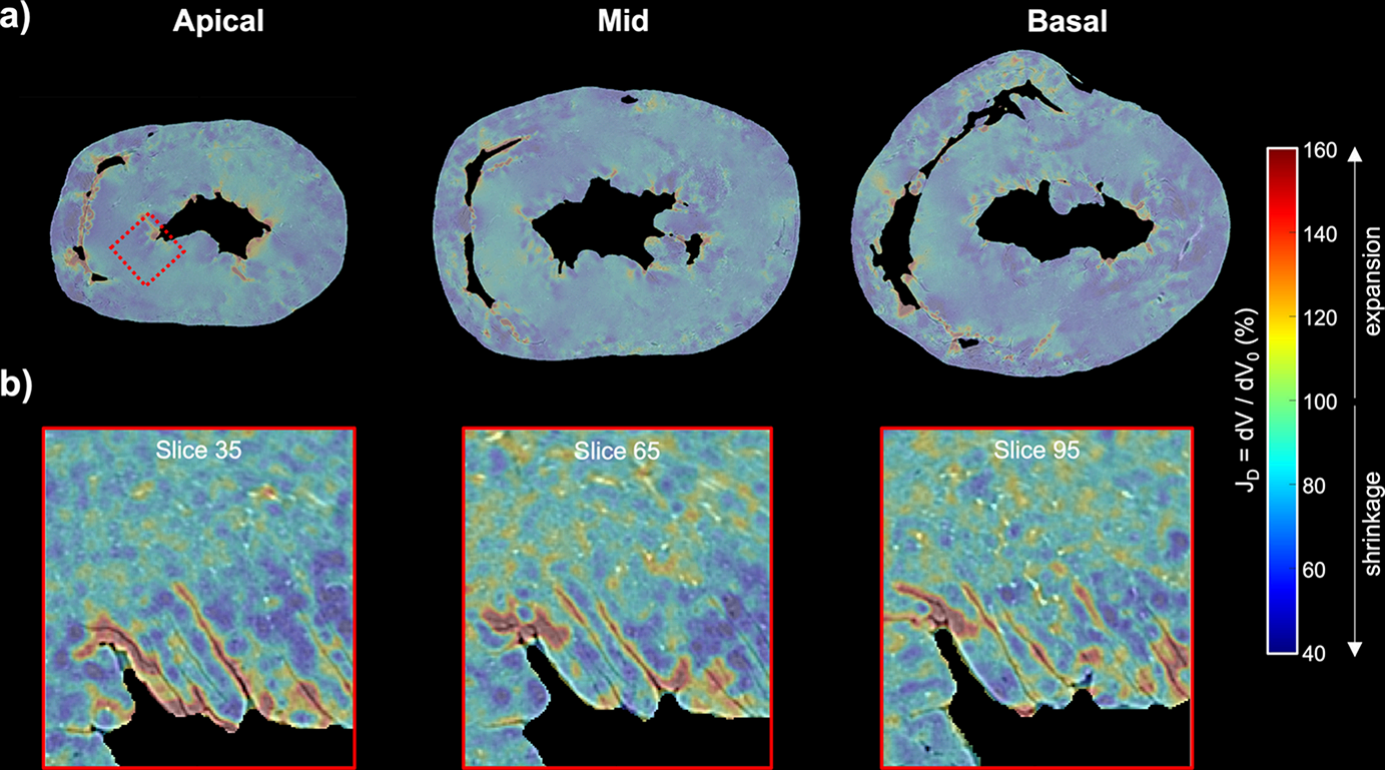
Quantification of the local myocardial volume changes due to ethanol dehydration. (*a*) Colour maps of the local Jacobian determinants of the deformation field, *J*_D_, at three levels from base to apex, obtained after the non-rigid registration between the heart before and 342 h after ethanol immersion. (*b*) Local *J*_D_ at three different image slices obtained after the non-rigid registration at full resolution within the ROI indicated with a dashed red rectangle. Reddish values indicated tissue expansion while blueish values indicated tissue shrinkage.

Looking at Fig. 3 ▸ one can realize that local deformation induced by ethanol dehydration is not uniform, since there are areas with a high degree of shrinkage (dark blue) and areas where tissue shrinkage is very low (turquoise/green). Values higher than 100% (expansion, since 100% corresponds to no volume changes) are observed along the spaces between myocardium and cavities, indicating that cavity space is increased due to tissue contraction.

### 3.3. Quantification of myocardial organization

**Figure 4:**
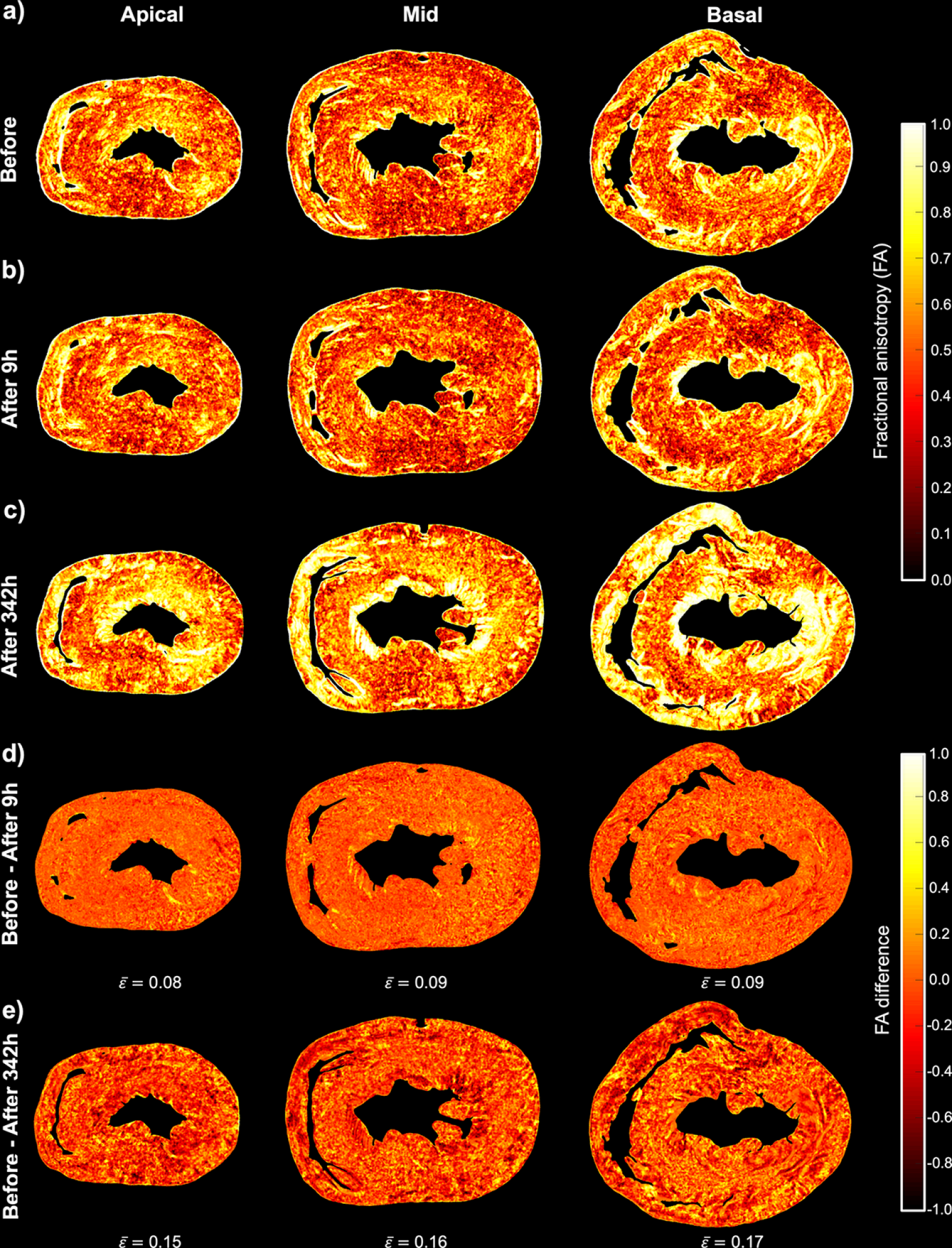
Quantification of myocardial organization via fractional anisotropy (FA). Local FA at three levels from base to apex of the rat heart (*a*) before, (*b*) 9 h after and (*c*) 342 h after ethanol immersion. Difference in local FA between the heart before and (*d*) 9 h after and (*e*) 342 h after ethanol immersion. The average FA difference (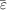) was calculated for each image slice.

Figs. 4 ▸ and 5 ▸ show colour maps of the local values of FA_ST_ and HA, respectively, for all three datasets: before, 9 h after and 342 h after ethanol immersion. Regarding FA_ST_, there is a significant increase of about 0.16 due to tissue dehydration 342 h after ethanol immersion, while differences are minimal between the heart before and 9 h after ethanol immersion (Fig. 4 ▸).

**Figure 5:**
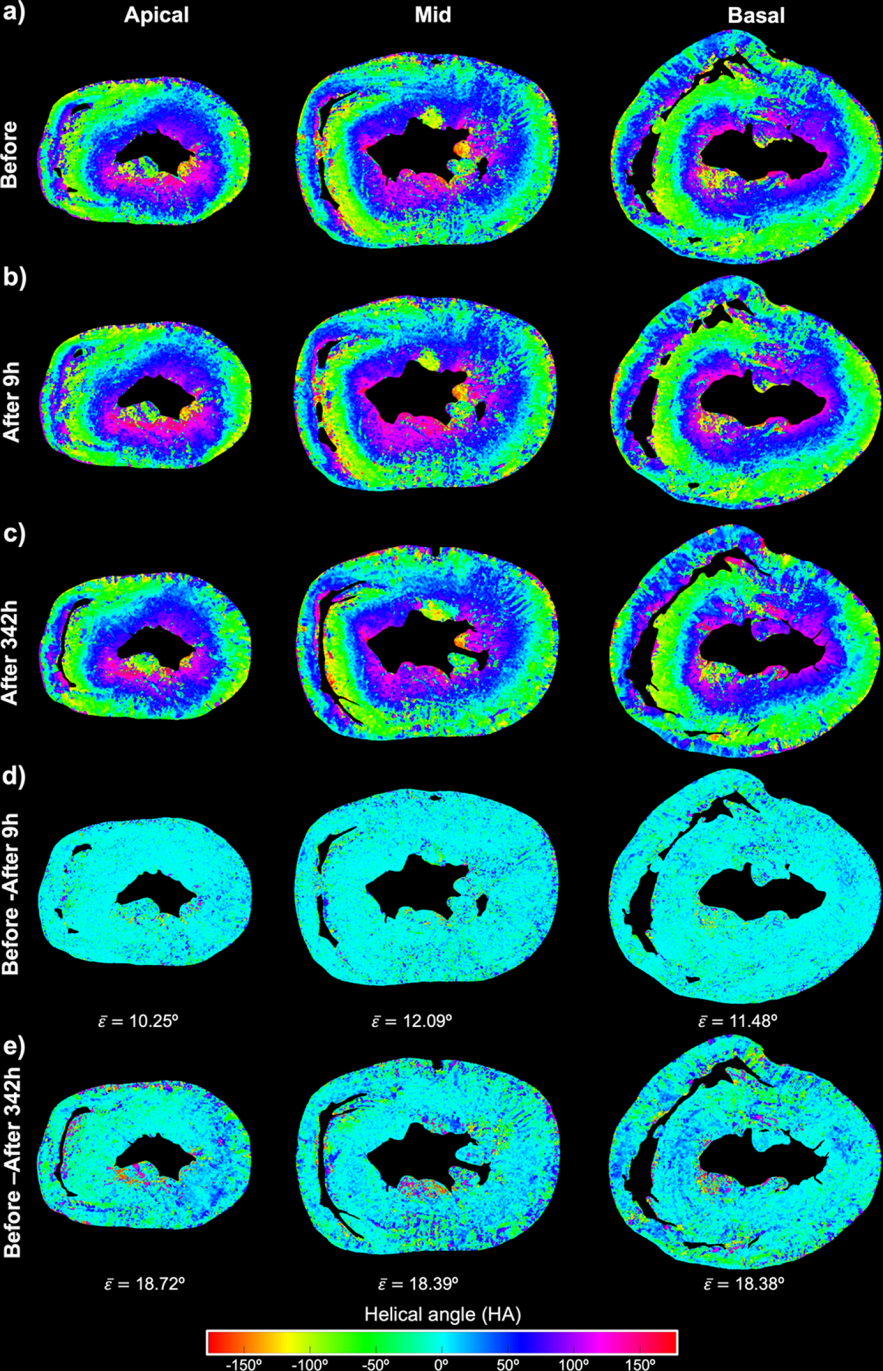
Quantification of local helical angle (HA) at three levels from base to apex of the rat heart (*a*) before, (*b*) 9 h after and (*c*) 342 h after ethanol immersion. Difference in local HA between the heart before and 9 h after (*d*) and 342 h after (*e*) ethanol immersion. The average HA difference (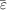) was calculated for each image slice.

**Figure 6:**
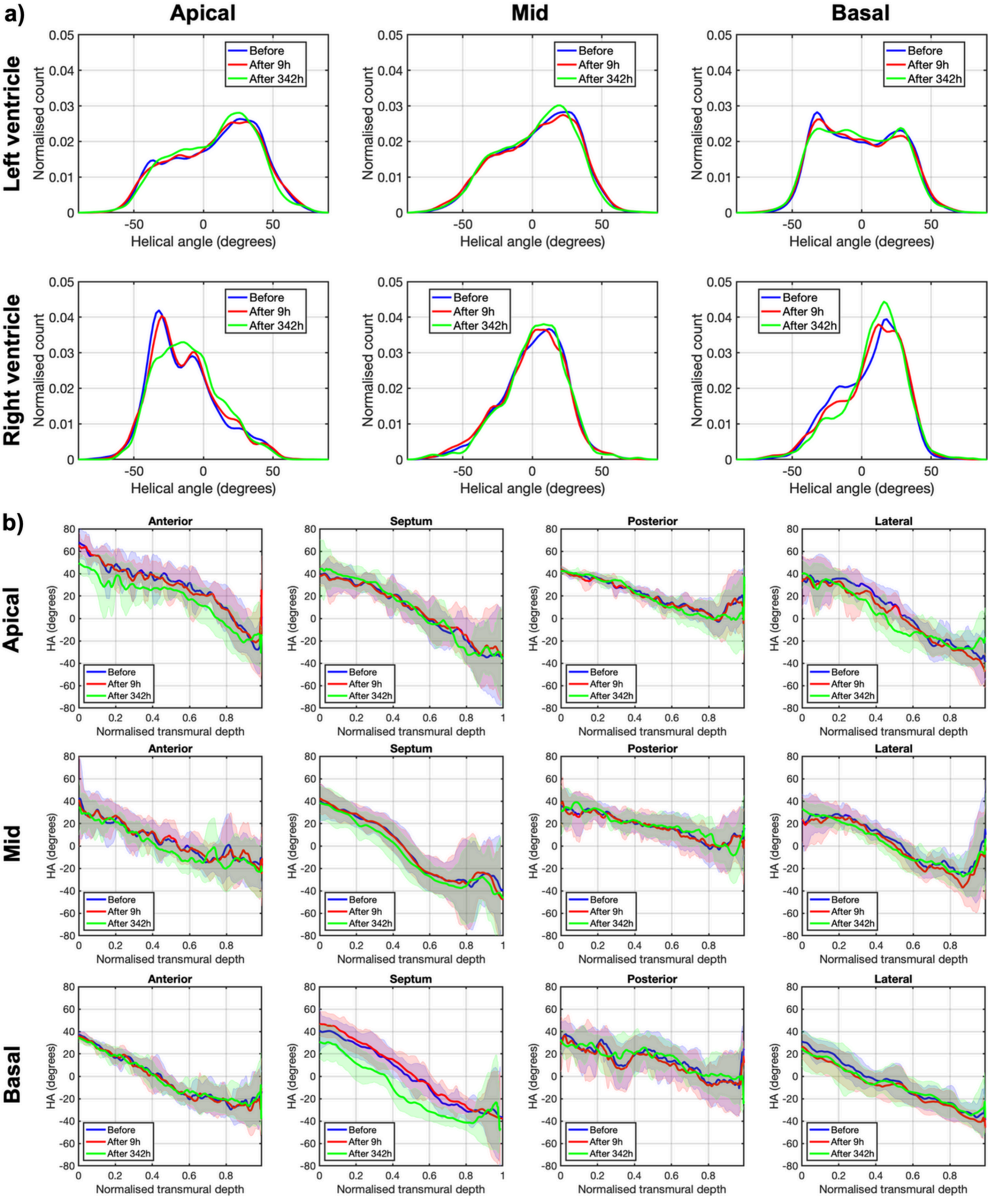
Quantification of myocytes aggregates orientation via helical angle (HA). (*a*) Histograms of HA in the left ventricle (LV) and in the right ventricle (RV) across the basal, mid-ventricular and apical slices of the rat heart before (blue), 9 h after (red) and 342 h after (green) ethanol immersion. (*b*) LV transmural profiles of HA across four LV segments (anterior septal, posterior and lateral) in the same three apico-basal slices of the rat heart before (blue), 9 h after (red) and 342 h after (green) ethanol immersion. Solid lines: sample mean. Shadows: ± standard deviation.

For HA, it can be observed that, while there are only small differences between the heart before and 9 h after ethanol immersion, the differences are much bigger (the average HA difference is about 18°; Fig. 5 ▸) between the heart before and 342 h after ethanol immersion. There are no differences neither in the HA transmural profiles nor HA distributions between the heart before and 9 h after ethanol immersion (Fig. 6 ▸; Table S1). However, while the slope and linearity coefficient of the transmural profile of HA remain unchanged, a significant decrease in the intercept of the transmural profile of HA is noticed in the heart 342 h after ethanol immersion (41.18 ± 9.59 versus 36.93 ± 9.17; *p* = 0.0015), being more pronounced in the basal septal and apical anterior walls (see Table 3 ▸ and Fig. 5 ▸).

Finally, while distributions of *C*_p_, *C*_s_ and *C*_l_ of the heart before and 9 h after ethanol immersion are very similar, an increase in planar anisotropy *C*_p_ together with the subsequent decrease in *C*_s_ and *C*_l_ is observed in the heart 342 h after ethanol immersion (see Fig. S3). This can be explained by the fact that, since the ventricular intramyocardial space between layers of myocytes, also known as cleavage planes, is increased due to tissue shrinkage, the laminar arrangement of myocytes becomes more evident.

## 4. Discussion

In the present study we have quantitatively shown that the use of ethanol dehydration in synchrotron X-PCI can improve image contrast at the expense of inhomogeneous tissue shrinkage, which has been shown to disrupt local myocardial organization. This has been demonstrated by the changes in the quantitative parameters commonly used to quantify myocardial organization such as HA and FA.

To our knowledge this is the first study that performs a detailed quantitative analysis of the local myocardial changes produced by ethanol dehydration. First, a rat heart was imaged with synchrotron-based X-PCI, before, 9 h after and 342 h after its immersion in 70% ethanol solution. Then, we quantified image contrast, global volume and myocardial thickness changes due to ethanol dehydration. In order to determine local deformation of the heart caused by ethanol dehydration, the related 3D tomographic datasets were registered by means of a non-rigid registration algorithm. The non-rigid registration was then used to recover the deformation fields, and the local relative volume change was computed. Finally, myocardial organization parameters such as FA and the orientation of myocytes aggregates were also computed and compared in the three tomographic datasets.

The global volume shrinkage was found to be 27.7% after long-term immersion of the heart in 70% ethanol. Moreover, we found a heterogeneous reduction of LV myocardial thickness of about 18.3 ± 6.7% on average, with maximum values of about 27% in the basal anterior and lateral and mid-anterior walls. Similar results were found by Patzelt *et al.* (2019 ▸) who evaluated three ethanol fixation protocols for heart and lung micro-CT imaging including fixed ethanol concentrations of 50% and 97% and four consequent ethanol baths of increasing concentrations. They reported a 21% volume shrinkage, high sample stiffness and small tissue ruptures due to fast dehydration in the heart after 168 h immersion in 97% ethanol. The authors concluded that fixation in a series of ascending ethanol concentrations provided the best results in terms of contrast enhancement and tissue preservation avoiding tissue ruptures. Similarly, Dudak *et al.* (2016 ▸) also tested three different ethanol fixation techniques (50%, 97% and series of increasing concentration 50–97%) in soft tissue murine organs and found very similar results in terms of contrast enhancement. However, they did not quantify the volume changes due to tissue dehydration. Holda *et al.* (2016 ▸) investigated the effect of different types of fixatives and concentrations on the dimensions of cardiac tissue and found that, after 168 h immersion in 85.5% ethanol, heart weight was significantly reduced by 16%. They also found a statistically significant but heterogeneous myocardial wall thickness reduction of up to 16% in the RV. On the other hand, Buytaer *et al.* (2014 ▸) quantified the total volume shrinkage of three tissue types: bone, muscle and nerve tissue, caused by four common specimen preparations: two water-based and two ethanol-based methods using the same dehydrating alcohol series (30, 60, 90, 100 and 100%, each for at least a day). To do that, they scanned the samples before (fresh) and after specimen preparation for light sheet fluorescence microscopy (LSFM) or micro-CT. They found that LSFM preparation of the samples, regardless of the tissue type, caused the most volume shrinkage compared with the other methods, with an average volume shrinkage for muscle of 56 ± 2%.

Most of the previous studies evaluated the global volume shrinkage produced by ethanol dehydration. The present study, however, determined the local volume changes induced by ethanol within the whole heart by means of a non-rigid registration method. The most important advantage of this technique is the possibility to analyse local shrinkage and deformations of different parts of the heart. Our results clearly show that shrinkage is anisotropic with areas with a high degree of shrinkage and areas where shrinkage is very low, or even where the tissue expands as illustrated in Fig. 3 ▸. The maximum expansion values were observed along the spaces between myocardium and cavities, explained by the increase of the cavity space due to tissue contraction. Schulz *et al.* (2011 ▸) used a similar approach to quantify the local deformation produced by formalin fixation in the brain. They found maximal local volume strain values of 22% and −24% due to formalin fixation. The maximal expansion values were mainly located at the ventricles which can be explained by the enlargement of the ventricles due to the lower pressure of the brain outside the cranium.

Myocardial walls are formed by myocytes that are aggregated together forming a complex 3D network surrounded by a matrix of connective tissue, showing predominant directions within the ventricular myocardium. The complex organization of these aggregates of myocytes within the myocardium determines the propagation of electrical waves as well as the force development within cardiac tissue. However, there is still some controversy regarding the fashion in which the individual cardiomyocytes are aggregated together (Anderson *et al.*, 2019 ▸). Several studies have suggested that myocytes are also grouped forming flattened structures of approximately four myocytes thick named: ‘sheets’, ‘sheetlets’, ‘laminar’ or ‘lamellar’ (Varray *et al.*, 2017 ▸; Mirea *et al.*, 2015 ▸; Wang *et al.*, 2019 ▸; Varray *et al.*, 2013 ▸; LeGrice *et al.*, 1995 ▸, 2005 ▸; Gilbert *et al.*, 2012 ▸). In this work we have shown that cleavage planes are more evident after long-term immersion of the heart in 70% ethanol, especially in the papillary muscles and endocardial surface as illustrated in Fig. 1 ▸ and Figs. S1 and S2. This is also illustrated by the increase in the planar anisotropy *C*_p_, and decrease in the spherical anisotropy *C*_s_ of the heart after long-term immersion in ethanol. Interestingly, most of the works that have described the existence of laminar structures within the myocardium have used ethanol as a fixative solution, as part of the tissue processing and/or to improve image contrast, or other contrast agents that also cause tissue shrinkage (Mirea *et al.*, 2015 ▸; Wang *et al.*, 2019 ▸; Varray *et al.*, 2017 ▸, 2013 ▸; LeGrice *et al.*, 1995 ▸, 2005 ▸; Gilbert *et al.*, 2012 ▸).

We have also shown that the heterogeneous deformation occurring as a consequence of tissue dehydration also had an impact on the 3D organization of the myocardium. After long-term immersion of the heart in ethanol we observed a significant increase of the FA_ST_ as well as significant changes in the HA. Our results suggest that when performing quantitative studies of myocardial organization it is very important that all the samples are processed in the same way, especially when comparing different groups, and that the possible effects of the use of chemical agents such as the non-uniform deformation of the tissue have to be taken into the account.

### 4.1. Limitations

The main limitation of the present study is the number of samples, since we have only performed the experiment in a single rat heart. However, we think that one sample was enough as a proof of principle to illustrate the effects of ethanol on myocardial tissue. Moreover, in this study we have also evaluated one protocol for tissue dehydration. Therefore, in order to optimize sample preparation, several ethanol protocols should be evaluated and compared, as well as different tissue types. In this way, study protocols can be optimized to tissue type and size, by maximizing contrast while minimizing tissue alterations.

## 5. Conclusions

In conclusion, we have quantitatively demonstrated that the use of ethanol as contrast agent in synchrotron X-PCI enhances image contract but the tissue deformation occurring as consequence of dehydration is not uniform, thus altering the 3D myocardial organization.

## 6. Related literature

The following references, not cited in the main body of the paper, have been cited in the supporting information: Pennec *et al* (1999 ▸); Thirion (1998 ▸).

## Supplementary Material

Supplementary Material. DOI: 10.1107/S1600577524010221/gui5002sup1.pdf

## Figures and Tables

**Table 1:** Quantification of changes in cardiac tissue contrast and image quality between all three datasets: before, after 9 h and after 342 h of ethanol treatment CNR: contrast to noise ratio; *C*: tissue contrast; SNR: signal-to-noise ratio

	Before	After 9 h	After 342 h
CNR	0.097 ± 0.028	0.195 ± 0.023	0.127 ± 0.015
*C* (%)	1.084 ± 0.178	2.804 ± 0.094	3.883 ± 0.251
SNR (dB)	27.17 ± 2.85	28.91 ± 1.17	24.15 ± 0.63

**Table 2:** Myocardial volume and left ventricular (LV) wall thickness measurements

	Before	After 9 h		After 342 h	
		Value	Change (%)	Value	Change (%)
Myocardial volume (cm^3^)	0.75	0.75	0	0.54	−27.69
LV wall thickness (mm)					
Basal anterior	3.19	3.15	1.30	2.34	−26.88
Basal septal	3.16	3.16	0.05	2.53	−19.98
Basal posterior	3.84	3.84	−0.03	3.13	−18.40
Basal lateral	3.10	3.05	1.69	2.27	−26.79
Mid-anterior	4.51	4.49	0.37	3.27	−27.48
Mid-septal	2.91	2.94	-0.78	2.56	−12.13
Mid-posterior	2.53	2.52	0.53	2.31	−8.90
Mid-lateral	3.82	3.78	0.89	2.99	−21.50
Apical anterior	2.77	2.77	−0.05	2.30	−16.95
Apical septal	3.07	3.07	0.03	2.80	−8.79
Apical posterior	2.44	2.43	0.41	1.98	−18.85
Apical lateral	2.80	2.79	0.28	2.43	−13.34
Mean ± STD	3.18 ± 0.60	3.17 ± 0.60	−0.39 ± 0.66	2.58 ± 0.39	−18.33 ± 6.65

**Table 3:** Parameters of the linear fitting (*y* = β_1_*x* + β_0_) of the HA transmural profile within the LV rat heart before and after 342 h of ethanol dehydration

	Slope: β_1_ (°)		Intercept: β_0_ (°)		Linearity (*R*^2^)	
	Before	After 342 h	Before	After 342 h	Before	After 342 h
Basal anterior	−75.45	−69.27	33.85	31.20	0.97	0.96
Basal septal	−94.87	−92.09	46.77	30.21	0.99	0.95
Basal posterior	−40.22	−33.67	33.92	31.91	0.83	0.85
Basal lateral	−69.48	−59.44	28.34	21.58	0.99	0.98
Mid-anterior	−54.87	−57.04	31.64	28.36	0.94	0.91
Mid-septal	−97.32	−96.79	43.62	39.51	0.95	0.95
Mid-posterior	−40.91	−36.87	36.83	36.88	0.96	0.94
Mid-lateral	−69.75	−70.97	36.49	34.63	0.93	0.98
Apical anterior	−66.13	−61.68	62.81	48.34	0.92	0.89
Apical septal	−82.47	−93.52	47.85	53.45	0.97	0.97
Apical posterior	−48.53	−55.82	43.50	45.78	0.98	0.97
Apical lateral	−86.29	−84.41	48.59	41.34	0.93	0.96
Mean ± STD	−68.8 ± 19.6	−67.63 ± 21.03	41.18 ± 9.59	36.93 ± 9.17	0.94 ± 0.04	0.94 ± 0.04
