# Supplementary material for: The use of ethanol as contrast enhancer in synchrotron X-ray phase-contrast imaging leads to heterogeneous myocardial tissue shrinkage: a case report
Source: J Synchrotron Radiat. 2025 Jan 1;32(Pt 1):200–9. doi: 10.1107/S1600577524010221 (PMC11708853; doi:10.1107/S1600577524010221)
Supplement: Supplementary file 1 [file s-32-00200-sup1.pdf]

JOURNAL OF SYNCHROTRON RADIATION

Volume 32 (2025)

Supporting information for article:

The use of ethanol as contrast enhancer in Synchrotron X-ray phase-contrast imaging leads to heterogeneous myocardial tissue shrinkage: a case report

Gabriel Bernardino, Àngels Calvet-Mirabent, Hector Dejea, Eduard Guasch, Anne Bonnin and Patricia Garcia-Canadilla

S1. Supplementary methods

Demons is an iterative registration algorithm introduced by Thirion (Pennec et al., 1999; Thirion, 1998). In this algorithm, each voxel of the moving image is subject to a force computed via the optical flow equation (inward force) proportional to the intensity difference between the voxels of both images, thus decreasing the difference between the two images. This force is then spatially regularised via a diffusion process (onward force). Symmetric force additive demons algorithm is a variation of the classical demons algorithm in which the forces are symmetric (Rogelj et al., 2006; Wang et al., 2005), i.e. the optical flow from the fixed to the moving images is also included to the inward force. At each step, the forces between the warped (using the currently estimated deformation field) moving and fixed image are computed, and the estimated deformation field is updated following these forces. Pennec et al. (Pennec et al., 1999) showed that the iterative process converges to the minima of an energy function, thus allowing to express the registration as a minimization problem that can be solved using standard optimization algorithms. We chose the minimization formulation that we solved using gradient descent.

S2. Supplementary Tables

Table S1 Parameters of the linear fitting ($y = \beta_1 \cdot x + \beta_0$) of the *HA* transmural profile within the left ventricle (LV) of the rat heart before and after 9h of ethanol dehydration.

	**Slope: β_1 (°)**		**Intercept: β_0 (°)**		**Linearity (R^2)**	
	**Before**	**After 9h**	**Before**	**After 9h**	**Before**	**After 9h**
**Basal Anterior**	-75.45	-74.69	33.85	33.06	0.97	0.97
**Basal Septal**	-94.87	-97.84	46.77	51.38	0.99	1.00
**Basal Posterior**	-40.22	-39.83	33.92	30.80	0.83	0.85
**Basal Lateral**	-69.48	-62.99	28.34	21.52	0.99	0.98
**Mid Anterior**	-54.87	-54.67	31.64	32.16	0.94	0.95
**Mid Septal**	-97.32	-97.06	43.62	44.01	0.95	0.94
**Mid Posterior**	-40.91	-39.26	36.83	35.61	0.96	0.97
**Mid Lateral**	-69.75	-75.06	36.49	35.41	0.93	0.93
**Apical Anterior**	-66.13	-67.77	62.81	62.15	0.92	0.94
**Apical Septal**	-82.47	-76.84	47.85	46.01	0.97	0.97
**Apical Posterior**	-48.53	-48.42	43.50	42.83	0.98	0.98
**Apical Lateral**	-86.29	-84.42	48.59	44.19	0.93	0.96
**Mean +/- STD**	-68.80 ± 19.60	-68.24 ± 19.96	41.18 ± 9.59	39.93 ± 10.76	0.95 ± 0.04	0.95 ± 0.04

S3. Supplementary Figures

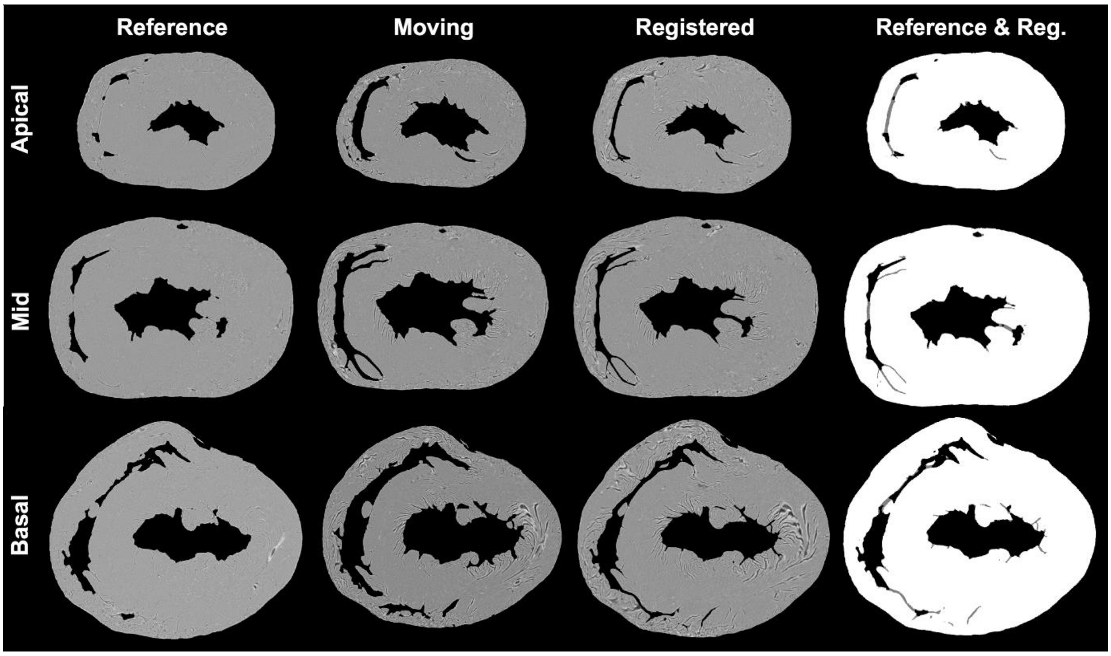

Figure S1 Results of the 3D whole heart non-rigid registration between the reference (before ethanol immersion) and moving (342h after ethanol immersion) datasets. Results are shown for three different apico-basal slices of the heart.

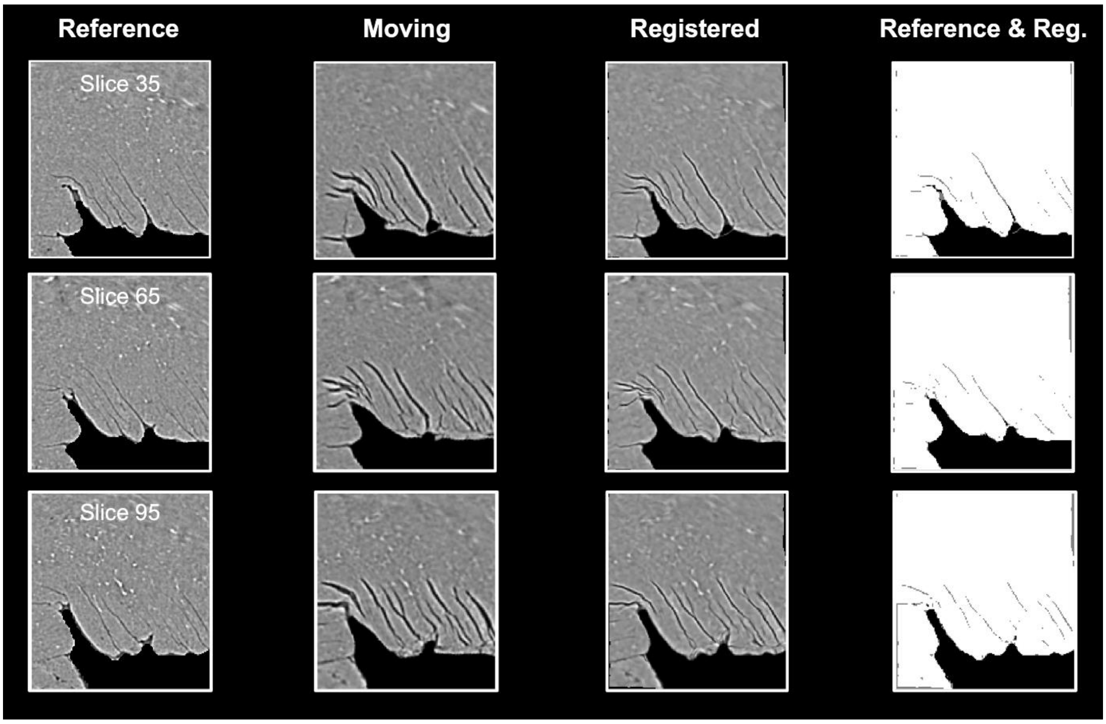

Figure S2 Results of the 3D non-rigid registration within a ROI located in the left ventricular endocardium, between the reference (before ethanol immersion) and moving (342h after ethanol immersion) datasets. Results are shown for three different slices of the ROI.

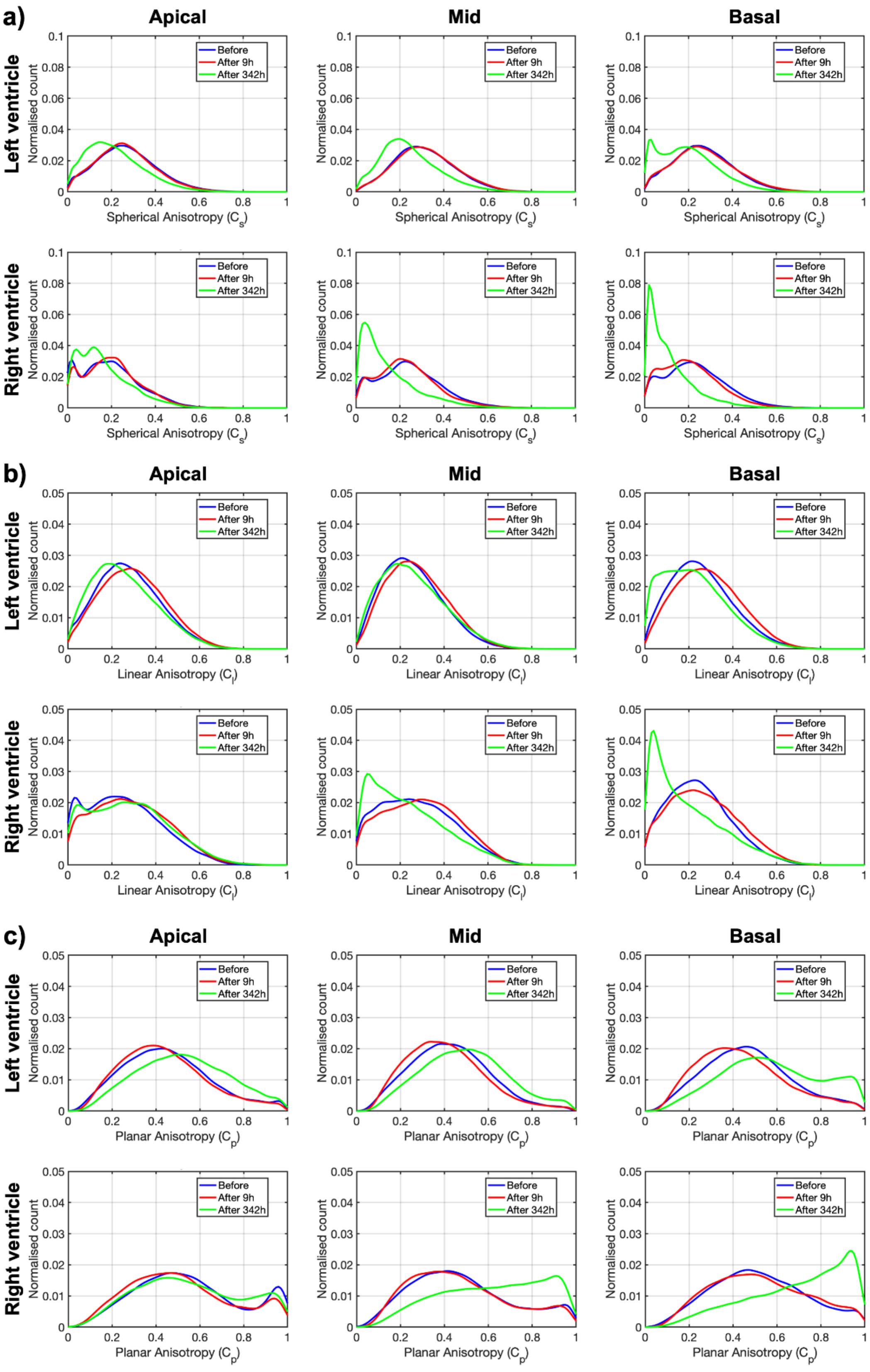

Figure S3 Distributions of the **(a)** spherical (C_s), **(b)** linear (C_l) and **(c)** planar anisotropy (C_p) in the left (LV) and right ventricles (RV) calculated at three different apico-basal image slices.

References

Pennec, X., Cachier, P., & Ayache, N. (1999). Understanding the "demon's algorithm": 3D non-rigid registration by gradient descent. *Lecture Notes in Computer Science (Including Subseries Lecture Notes in Artificial Intelligence and Lecture Notes in Bioinformatics)*, *1679*, 597–606. https://doi.org/10.1007/10704282_64

Rogelj, P., Kovacic, S., & Kovačič, S. (2006). Symmetric image registration. *Medical Image Analysis*, *10*(3 SPEC. ISS.), 484–493. https://doi.org/10.1016/j.media.2005.03.003

Thirion, J.-P. (1998). Image matching as a diffusion process: an analogy with Maxwell's demons. *Medical Image Analysis*, *2*(3), 243–260. https://doi.org/10.1016/s1361-8415(98)80022-4

Wang, H., Dong, L., O'Daniel, J., Mohan, R., Garden, A. S., Ang, K. K., Kuban, D. A., Bonnen, M., Chang, J. Y., & Cheung, R. (2005). Validation of an accelerated "demons" algorithm for deformable image registration in radiation therapy. *Physics in Medicine and Biology*, *50*(12), 2887–2905. https://doi.org/10.1088/0031-9155/50/12/011
